# Revision Total Hip Arthroplasty in Solid Organ Transplant Patients: A Propensity Score-Matched Cohort Study for Aseptic and Infected Revisions

**DOI:** 10.1016/j.artd.2021.10.007

**Published:** 2022-01-20

**Authors:** Alex Upfill-Brown, Christopher M. Hart, Peter P. Hsiue, Kadarius Burgess, Clark J. Chen, Amir Khoshbin, Christos Photopoulos, Alexandra I. Stavrakis

**Affiliations:** a Department of Orthopaedic Surgery, David Geffen School of Medicine at UCLA, Los Angeles, CA, USA; b Division of Orthopaedic Surgery, University of Toronto, Toronto, ON, Canada; c Cedars-Sinai Kerlan-Jobe Institute, Los Angeles, CA, USA

**Keywords:** Revision total hip arthroplasty, Solid organ transplant, Surgical outcomes, Prosthetic joint infection

## Abstract

**Background:**

Previous studies have demonstrated that solid organ transplant (SOT) patients undergoing primary total hip arthroplasty (THA) are at an increased risk of postoperative complications. The purpose of this study is to use a large, national database to investigate revision THA (rTHA) outcomes in SOT patients.

**Methods:**

Nationwide Readmissions Database (NRD) from 2010-2018 was used, and ICD-9 and ICD-10 codes were used to identify all patients who underwent rTHA, including those with history of SOT. Propensity score matching (PSM) was used to analyze rTHA outcomes in SOT patients comparted to matched controls. Separate analysis performed for patients undergoing rTHA for prosthetic joint infection (PJI) vs other causes.

**Results:**

A total of 414,756 rTHA, with 1837 of those being performed in SOT patients, were identified. Of these, 65,961 and 276 were performed for PJI in non-SOT and SOT patients, respectively. For non-PJI patients, SOT patients had higher 90-day all-cause readmission rates (24.0% vs 19.4%, *P* = .03) but lower rate for readmission related to rTHA (6.0% vs 9.2%, *P* = .03), but no difference readmission for specific rTHA complications, mortality (0.6% vs 1.3%, *P* = .20), or revision rTHA. Of PJI patients, SOT patients had no difference in overall 90-day readmission (38.6 vs 31.3%, *P* = .280), readmission for specific rTHA complications, re-revision, or mortality (4.7% vs 6.0%, *P* = .63).

**Conclusions:**

SOT patients undergoing rTHA for aseptic reasons are higher risk of overall readmission but lower risk of readmission related to rTHA than appropriately matched controls. SOT PJI patients undergoing had similar rates of readmission, mortality, and revision surgery compared to matched non-SOT PJI patients.

## Introduction

Total hip arthroplasty (THA) has long been established as a safe and effective treatment for hip arthritis with significant benefits for appropriately selected patients [1]. Given the advances in surgical technique, perioperative management, and low overall complications rates, the rate of THAs in medically complex patients is also expected to grow. Researchers predict the number of THA procedures performed annually in the United States to reach 900,000 by 2030, and 1.23 million by the year 2060. During this same time period, they also predict the number of rTHA performed to increase by 219% to 110,000 annually [2].

At the same time, survivorship in patients who have undergone solid organ transplant (SOT) has steadily improved in part because of improvements in surgical technique and postoperative immunosuppression and surveillance protocols [3]. As SOT patients live longer, the incidence of symptomatic arthritis is likely to increase, leading to an increased demand for both primary and revision joint replacement procedures [4,5]. SOT patients are also at higher risk to develop femoral head avascular necrosis, patients with renal transplants, in particular, undergo THA at 5-8 times the rate of the general population [6].

In primary joint replacement, patients with a history of SOT have been shown to have a higher rate of morbidity and mortality [7,8]. It is less clear how SOT patients do after revision THA (rTHA). A retrospective case series of 30 patients found an elevated risk for re-revision, particularly for prosthetic joint infection (PJI) in patients with a history of SOT [9]. More recently, Labaran et al. performed a study using a national administrative database and identified 661 rTHAs performed specifically in renal transplant recipients and found an increased risk of 90-day hospital readmission, 1-year septicemia, and 1-year mortality compared to matched controls [10]. Unfortunately, there are no currently available data in the literature which stratify outcomes based on aseptic vs infected revisions, which has been shown to influence the risk for 30-day complications after arthroplasty [11,12]. Furthermore, no studies have investigated outcomes more broadly for all types of SOT patients.

We present a propensity score matched cohort study using a large national administrative database to compare the outcomes of aseptic and infected revisions in patients with a history of SOT to matched controls. We hypothesize that SOT patients undergoing rTHA will have a higher length of stay (LOS) and increased rate perioperative complications.

## Material and methods

The study cohort was identified from the Nationwide Readmissions Database (NRD) over a 9-year study period (2010–2018). The NRD is a nationally representative database developed and validated through a federal-state-industry partnership sponsored by the Agency for Healthcare Research and Quality. It is based on 22 state inpatient databases that track patients across multiple hospitals. Approximately 51.2% of the US population and 49.3% of all US hospitalizations were sampled in a stratified algorithm, designed to allow for estimation of nationally representative statistics. Available variables include demographic data, diagnoses, procedures, cost, LOS, and hospital characteristics. Because the NRD database has been sufficiently deidentified, this study was deemed exempt by the institutional review board at our institution.

Patients older than 18 years who were admitted for rTHA were considered for this study. Patients were identified using the International Classification of Diseases, ninth and tenth revision, (ICD-9 and ICD-10) procedure codes (Table 1). For ICD-10 procedure codes, patients required either a revision code or a relevant removal and replacement code as described [13]. Patients were separated into groups based on whether or not they had a diagnosis of SOT (Table 1). Indication for rTHA was determined based on associated ICD-9 and ICD-10 diagnostic codes (Appendix Table 1). All subsequent readmissions were considered for these groups. Baseline comorbidity was quantified using the Elixhauser Comorbidity Index (ECI), a composite score of 30 comorbid conditions using all admission diagnoses and the comorbidity package in R. [14] Higher ECI scores corresponded to greater burden of comorbid conditions. ECI score component variables were also extracted.

**Table 1:** Diagnostic and procedural codes used to identify patients with a history of SOT and rTHA procedure types.

Transplant history	Type	ICD-9	ICD-10
	Kidney	V42.0	Z94.0
	Liver	V42.7	Z94.4
	Heart	V42.1	Z94.1, Z94.3
	Lung	V42.6	Z94.2, Z94.3
	Pancreas	V42.83	Z94.83
Component revised	Operation	ICD-9	ICD-10
hip (both)	Revision	00.70, 81.53	0SW908Z, 0SW90EZ, 0SW90JZ, 0SWB08Z, 0SWB0EZ, 0SWB0JZ
	Removal		0SP908Z, 0SP90EZ, 0SP90JZ, 0SPB08Z, 0SPB0EZ, 0SPB0JZ
	Replacement		0SR9019, 0SR901A, 0SR901Z, 0SR9029, 0SR902A, 0SR902Z, 0SR9039, 0SR903A, 0SR903Z, 0SR9049, 0SR904A, 0SR904Z, 0SR9069, 0SR906A, 0SR906Z, 0SR90EZ, 0SR90J9, 0SR90JA, 0SR90JZ, 0SRB019, 0SRB01A, 0SRB01Z, 0SRB029, 0SRB02A, 0SRB02Z, 0SRB039, 0SRB03A, 0SRB03Z, 0SRB049, 0SRB04A, 0SRB04Z, 0SRB069, 0SRB06A, 0SRB06Z, 0SRB0EZ, 0SRB0J9, 0SRB0JA, 0SRB0JZ
Femur	Revision	00.72	0SWR0JZ, 0SWS0JZ
	Removal		0SPR0JZ, 0SPS0JZ, 0SP908Z, 0SP90EZ, 0SP90JZ, 0SPB08Z, 0SPB0EZ, 0SPB0JZ
	Replacement		0SRR019, 0SRR01A, 0SRR01Z, 0SRR039, 0SRR03A, 0SRR03Z, 0SRR0J9, 0SRR0JA, 0SRR0JZ, 0SRS019, 0SRS01A, 0SRS01Z, 0SRS039, 0SRS03A, 0SRS03Z, 0SRS0J9, 0SRS0JA, 0SRS0JZ
Acetabulum	Revision	00.71	0SWA0JZ, 0SWE0JZ
	Removal		0SPA0JZ, 0SPE0JZ, 0SP908Z, 0SP90EZ, 0SP90JZ, 0SPB08Z, 0SPB0EZ, 0SPB0JZ
	Replacement		0SRA009, 0SRA00A, 0SRA00Z, 0SRA019, 0SRA01A, 0SRA01Z, 0SRA039, 0SRA03A, 0SRA03Z, 0SRA0J9, 0SRA0JA, 0SRA0JZ, 0SRE009, 0SRE00A, 0SRE00Z, 0SRE019, 0SRE01A, 0SRE01Z, 0SRE039, 0SRE03A, 0SRE03Z, 0SRE0J9, 0SRE0JA, 0SRE0JZ
Isolated liner	Revision	00.73	0SU909Z, 0SUA09Z, 0SUB09Z, 0SUE09Z, 0SUR09Z, 0SUS09Z

The primary outcomes of interest include 90-day and 180-day mortality, readmission rates, as well as readmission stratified by indication, and re-revision THA. Secondary outcomes included complications during index hospitalization. ICD-9 and ICD-10 codes were used to identify cardiac complications, myocardial infarction, cerebrovascular accident, respiratory complications, pneumonia (PNA), pulmonary embolism (PE), other pulmonary complications, deep vein thrombosis (DVT), acute kidney injury, wound complications, postoperative blood transfusions, or any in-hospital complications (Appendix Table 2).

Propensity score matching (PSM) was performed to compare relative risks (RR) of re-admissions and complications in transplant and non-transplant patients. [15] A propensity score multivariate logistic regression model was created using patient age, sex, ECI, hospital type, hospital size, insurance status, and zip code income quartile. Specific medical comorbidities were also in the model, including history of CHF, cardiac arrhythmia, pulmonary hypertension, chronic pulmonary disease, essential hypertension, diabetes, obesity, coagulopathy, solid tumor, and alcohol abuse. Patients undergoing rTHA for infection were analyzed separately from those undergoing rTHA for other reasons (ie, loosening, instability). Propensity scores were used to match transplant patients to nontransplant patients at a ratio of 1:3 with replacement improved balance using the MatchIt package in R [16]. Relative risk was estimated using weighted logistic regression.

All result sample sizes represented national estimates taking into account the NRD’s stratified 2-stage cluster design incorporating individual discharge-level weights. Descriptive analysis was used to describe both baseline characteristics and outcome parameters within each comparison group. Categorical variables are compared using the chi-squared statistic, except when individual cell counts were less than 10, in which case the Fisher exact test was used. Continuous variables were reported using mean, 95% confidence interval (CI), and *P* values and were compared using the student t-test after ensuring normal distributions. For skewed distributions, continuous variables are presented as median (interquartile range) and the Wilcoxon rank-sum test. All tests were unpaired with a significance level defined as a 2-tailed P of 0.05. Statistical analyses were performed using R 3.6.0 (R Foundation for Statistical Computing, Vienna, Austria).

## Results

### Baseline characteristics

A total of 414,756 patients underwent rTHA during the study period, 412,919 nontransplant patients and 1837 transplant patients (Table 2). Of these, 65,961 (16.0%) and 276 (15.0%) were performed for infection in nontransplant and transplant patients, respectively (*P* = .274). Renal transplant patients were the most common (67.3%), followed by liver (24.3%) and heart (6.6%) (Table 3). Transplant patients tended to be younger than other rTHA patients (mean 58.7 vs 67.2 years, *P* < .001). Transplant patients were less likely to be female (41.5% vs 56.9%, *P* < .001). They also tended to have a higher baseline level of medical comorbidity (ECI mean 3.58 vs 2.29, *P* < .001). Transplant patients were more likely to undergo rTHA at an urban teaching hospital (80.8% vs 67.1%, *P* < .001) and at a large hospital (65.0% vs 56.0%, *P* < .001).

**Table 2:** Baseline characteristics of rTHA transplant and nontransplant patients identified over study period.

Variable	Non-SOT	SOT	*P*
	n = 412919	n = 1837	
Sex, female	234791 (56.9%)	762 (41.5%)	<.001
Age			
Mean	67.2	58.7	<.001
<60	112534 (27.3%)	842 (45.9%)	<.001
60-75	182101 (44.1%)	888 (48.4%)	
>75	118284 (28.6%)	106 (5.8%)	
Indication			
PJI	65961 (16%)	276 (15%)	.007
Fracture	38474 (9.3%)	107 (5.8%)	
Instability	78796 (19.1%)	361 (19.7%)	
Loosening	98628 (23.9%)	523 (28.5%)	
Other	131020 (31.7%)	569 (31%)	
ECI, mean	2.29	3.58	<.001
LOS, mean	5.23	5.92	.005
Payer			
Medicaid	18914 (4.6%)	60 (3.3%)	.12
Medicare	265609 (64.4%)	1236 (67.3%)	
Private	112621 (27.3%)	506 (27.5%)	
Other	12186 (3%)	30 (1.6%)	
Income, quartile			
0-25%	89723 (22.1%)	357 (19.7%)	.50
25-50%	105896 (26%)	471 (25.9%)	
50-75%	107195 (26.4%)	489 (26.9%)	
75-100%	103865 (25.5%)	499 (27.5%)	
Hospital type			
Rural	24662 (6%)	61 (3.3%)	<.001
Urban non-teaching	111070 (26.9%)	291 (15.8%)	
Urban teaching	277186 (67.1%)	1484 (80.8%)	
Hospital size			
Large	231115 (56%)	1194 (65%)	<.001
Medium	99628 (24.1%)	355 (19.3%)	
Small	82175 (19.9%)	287 (15.6%)

**Table 3:** Number of transplant patients by type of transplant.

	n	%
All Transplants	1837	
Kidney	1237	67.3%
Liver	447	24.3%
Heart	122	6.6%
Lung	66	3.6%
Pancreas	56	3.0%

**Table 4:** Propensity score matched analysis of probability of 90-day readmission and subsequent re-revision THA for non-PJI patients.

Complication	SOT patients	Matched controls	Relative risk	95% CI	*P* value
Any readmission	24.0%	19.4%	1.24	(1.02, 1.51)	.028
Related readmission	6.0%	9.2%	0.66	(0.44, 0.97)	.034
PJI	1.4%	3.9%	0.36	(0.16, 0.8)	.011
Instability	3.4%	4.4%	0.77	(0.45, 1.33)	.345
Loosening	0.8%	0.5%	1.71	(0.48, 6.19)	.406
Fracture	0.6%	1.1%	0.56	(0.16, 1.99)	.367
Re-revision THA	3.2%	5.5%	0.59	(0.34, 1)	.050
Mortality	0.6%	1.3%	0.45	(0.13, 1.55)	.202

### Infected vs noninfected patients

Univariate analysis of differences in outcomes of infected and noninfected rTHA patients showed a significant difference for SOT and non-SOT patients in the outcomes of interest. SOT patients undergoing rTHA for infection had a significantly longer LOS (10.2 vs 5.2 days, *P* < .001), a higher likelihood of readmission (35.3% vs 23.7%, *P* = .03) and a higher risk of subsequent revision surgery (11.6% vs 3.2%, p0.002) compared to those revised for non-infectious indications. Non-SOT patients undergoing rTHA for infection had a significantly longer LOS (9.6 vs 4.5 days, *P* < .001), higher likelihood of readmission (36.3% vs 16.8%, *P* < .001) and a higher likelihood of subsequent revision (16.6% vs 5.4%, *P* < .001) compared to those revised for non-infectious indications.

### PSM analysis of Non-PJI patients

For aseptic patients, SOT patients were more likely to be re-admitted for any reason within 90 days of surgery (20.4% vs 19.4%, *P* = .028) but less likely to be admitted with a diagnosis related to RTHA failure compared to matched controls (6.0% vs 9.2%, *P* = .034, Table 4). SOT patients were less likely to be readmitted with a diagnosis of PJI compared to matched controls (1.4% vs 3.9%, *P* = .01). There was no difference between SOT patients and matched controls regarding readmission for complications secondary to loosening, instability or fracture (Table 4). Compared to matched controls, SOT patients had a lower rate of 90-day overall re-revision THA with the difference approaching statistical significance (3.2% vs 5.5%, *P* = .050). There was no difference in 90-day mortality between SOT and matched non-transplant patients undergoing rTHA for aseptic causes (0.6% vs 1.3%, *P* = .20).

SOT patients had a lower rate of any complication (42.0% vs 48.3%, *P* = .007) and a lower risk of receiving post-operative blood transfusions (28.3% vs 36.4%, *P* < .001, Appendix Table 3) comparted to controls. There were no differences between groups with regard to cardiac, pulmonary, renal, DVT, PE and wound complications, specifically. The rates of PE and DVT were also not significantly different between groups. The LOS was significantly shorter for SOT patients comparted to controls (5.0 vs 6.0 days, *P* < .001).

### PSM analysis of PJI patients

For infected patients, there was no difference in overall 90-day readmission rate (36.8% vs 40.3%, *P* = .54), readmission related to RTHA, or for complications secondary to loosening, infection, instability, and fracture (Table 5). The rate of revision surgery was not different between groups at 90 days after RTHA (12.3% vs 17.9%, *P* = .19). SOT patients had a similar rate of 90-day mortality compared to matched patients (4.7% vs 6.0%, *P* = .63).

**Table 5:** Propensity score matched analysis of probability of 90-day readmission and subsequent re-revision THA for PJI patients.

Complication	SOT patients	Matched controls	Relative risk	95% CI	*P* value
Any readmission	36.8%	40.3%	0.91	(0.68, 1.22)	.538
Related readmission	17.0%	20.1%	0.84	(0.52, 1.37)	.487
PJI	13.2%	18.2%	0.72	(0.42, 1.26)	.247
Instability	2.8%	1.9%	1.50	(0.37, 6.09)	.567
Loosening	0.9%	0.9%	1.00	(0.1, 9.98)	.999
Fracture	0.0%	0.9%	0.00	(0, Inf)	.994
Re-revision THA	12.3%	17.9%	0.68	(0.39, 1.21)	.189
Mortality	4.7%	6.0%	0.79	(0.3, 2.1)	.633

There was no difference in index hospitalization wound complications (9.1% vs 9.6%, *P* = .87, Appendix Table 4) or postoperative blood transfusions (37.8% vs 45.0%, *P* = .15). There were no differences between groups in all complications or cardiac, pulmonary, or renal complications. The rates of PE and DVT were not significantly different between groups. The LOS was significantly shorter in the SOT group than that in the matched control group (10.0 vs 14.2 days, *P* = .01).

## Discussion

Over the past several decades, outcomes after SOT have continued to improve, and patients are living longer following transplantation [3]. It has been estimated that between 1987 and 2012, SOT resulted in 2.3 million life-years saved [17]. However, complications from immunosuppression, graft failure, and infection, among others, have been well documented in the transplant literature [18]. Arthroplasty surgery, and in particular revision arthroplasty, is also associated with risk, and the perioperative complications correlate in part with patient preoperative morbidity [19,20]. As patients with a history of both SOT and THA live longer, the need for rTHA will increase, highlighting the importance of anticipating risk to optimize outcomes. Furthermore, the indication for revision influences postoperative outcomes, with patients undergoing revision for infection generally faring worse. [12] For this reason, it is important to stratify rTHA outcomes by indication, specifically for aseptic causes vs infection.

Herein we present a propensity score matched cohort study using a large nationwide registry comparing outcomes of rTHA between SOT patients and matched controls for both aseptic and infected revisions. Rates of readmission and revision rTHA were significantly higher in PJI SOT and non-SOT patients than those in aseptic SOT and non-SOT patients, respectively. For patients undergoing aseptic rTHA, we found that patients with a history of SOT had an increased rate of overall readmission but a lower rate of readmission related to rTHA, including infection. Surprisingly, SOT patients had a lower rate of readmission for PJI than non-SOT patients. SOT patients also had lower rates of any complication or blood transfusion after rTHA compared to non-SOT patients. The lower rate of 90-day readmission related to rTHA and borderline lower rate of re-revision in the SOT cohort may reflect surgeon hesitancy to proceed with revision because of perceived increased risk in this population. The similar rate of hospital complications may reflect adequate matching between the cohorts based on demographics and comorbidities such as tobacco use, BMI, and diabetes mellitus status, which are known to be independent risk factors for post-operative medical complications, including SSIs. Labaran et al. reported no difference in 90-day major medical complications in patients with history of renal transplant undergoing revision arthroplasty compared to matched controls. [10] In a single institution review, Brown et al. also found no difference in peri-operative complications comparing SOT patients and matched controls who underwent primary total joint arthroplasty. [21]

Prior studies on rTHA in SOT patients are limited. Labaran et al. investigated outcomes after revision hip and knee arthroplasty in renal transplant patients using a large Medicare database from 2005 to 2015 [10]. In a matched analysis they found that renal transplant patients (total 661 patients) had an increased 90-day readmission (27.8% vs 23.2%), 90-day septicemia and 1-year mortality (6.8% vs 2.3%) rates than matched non-transplant patients; they found no difference in LOS or 1-year infection rates between group. They did not analyze re-operations or re-admissions for specific rTHA complications. In comparison, we similarly found a higher rate of 90-day readmission in SOT patients undergoing aseptic rTHA but a lower rate of readmission for reasons related to rTHA, with no differences in readmission or revision surgery for PJI rTHA patients. We found no difference in 90-day mortality for SOT patients undergoing rTHA for PJI specifically (4.7% vs 6.0%) or for other causes (0.6% vs 1.3%). In a single institution case series of 9 rTKA patients, Ledford et al. described outcomes in 30 SOT patients undergoing rTHA, of which 3 underwent re-revision for PJI and 3 for instability (total of 6, or 20%) at a mean of 2.1 years [9]. This is roughly comparable to the 90-day rate of re-operation following rTHA for PJI (12.3%) in SOT patients in this study. We found the rate of re-operation following rTKA for other reasons to be substantially lower (3.2%). The LOS in their study (5.0 days) is similar to that in this study for non-PJI patients (5.6 days).

The roughly similar rates of complications between SOT patients and matched controls in this study may be surprising to some, especially with regard to subsequent infection and mortality. PJI is the most feared complication following arthroplasty in SOT patients given the hypothesized higher risk posed by the use of chronic immunosuppressive medications in these patients. However, across surgical specialties, in comparative studies of elective surgical procedures adjusting for underlying medical comorbidity, SOT patients have similar rates of post-operative infections and wound complications [22,23]. Prior studies on complications following TJA in SOT patients have also not conclusively shown an increased risk [5,[24], [25], [26], [27]]. The reasons for this may be threefold. First, immunosuppression is not unique to SOT—many different types of patients are on immunosuppressive medications or immunosuppressed as a consequence of chronic disease [28]. Second, SOT patients often have higher levels of medical literacy and access to care than average [[29], [30], [31], [32]]. Finally, SOT patients are more likely to be treated at specialized academic centers, and consequently, as we find in this study, are more likely to receive their arthroplasty care at these same institutions. These studies suggest that although SOT patients’ immunosuppressive regimens may pose additional risk, some of that may be offset by increased access to specialized care and ease of navigating the healthcare system. Indeed, we find lower rates or readmission for infection in SOT patients undergoing aseptic rTHA, and no difference in readmission or revision surgery for SOT patients undergoing rTHA for PJI.

To our knowledge, this is the first report on rTHA in SOT patients which stratifies by all-cause aseptic indications and infection. Several authors have previously highlighted an increased mortality in septic vs aseptic revisions [21,[33], [34], [35]]. For both SOT and nontransplant patients, we found worse outcomes in patients undergoing revision for infection, including longer LOS, as well as higher rates of 90-day readmission and 90-day re-revision. The primary advantages of this study are the large sample size and stratified outcome analysis based on indication for revision. To date, only one other report has used a national administrative database to analyze outcomes of revision arthroplasty in transplant patients [10], but the number of patients analyzed was smaller and aseptic and infected revisions were considered jointly. We believe this may obscure important differences when considering the post-operative risks faced by this unique group of patients.

There are several limitations to this study. First, we recognize the inherent weaknesses in a large database study including potential for errors in coding and data entry. It is possible that some readmission events were missing from the NRD, biasing complication estimates downward in this study compared to single institution studies. Furthermore, the NRD allows for the analysis of short-term outcomes and therefore likely underestimates the true incidence of long-term complications after rTHA. Important clinical outcomes such as functional status, patient-reported outcome measures (PROMs), and pain scores are not recorded in the NRD. Despite controlling for demographic variables and comorbidities in our multivariate PSM analysis, there are some important confounding factors for which we were not able to control. Important surgical factors, including surgical complexity, were not available for analysis. Finally, information regarding surgical details such as implants used, procedure duration, intraoperative complications, and blood loss, was unavailable in the NRD.

## Conclusion

In the present study, we find that SOT alone is not consistently associated with increased risks after rTHA despite the increased medical comorbidity associated with transplantation. We found a higher overall readmission rate but a lower readmission rate related to rTHA for SOT patients undergoing aseptic rTHA. Rates of 90-day mortality and revision rTHA for both aseptic and infected revisions were similar between SOT patients and matched controls. Index hospital complications were lower overall in SOT patients than those in matched controls for both aseptic and infected rTHA. This suggests that transplant patients undergoing rTHA have no greater risk for complications and/or mortality at short-term follow-up (90 days) when compared to matched nontransplant patients. Access to specialist academic medical centers for SOT patients may offset the increased risk associated with immunosuppressive medication.

## Conflicts of interest

The authors declare that they have no known competing financial interests or personal relationships that could have appeared to influence the work reported in this article.

## Figures and Tables

**Appendix Table 1:** Categorized rTHA diagnostic codes for ICD-9 and ICD-10.

Group	ICD-9	ICD-10	ICD10 Text
Infection	996.66	T84.5	Infection and inflammatory reaction due to internal joint prosthesis
		T84.50	Infection and inflammatory reaction due to unspecified internal joint prosthesis
		T84.51	Infection and inflammatory reaction due to internal right hip prosthesis
		T84.52	Infection and inflammatory reaction due to internal left hip prosthesis
		T84.59	Infection and inflammatory reaction due to other internal joint prosthesis
	996.69	T84.7	Infection and inflammatory reaction due to other internal orthopedic prosthetic devices, implants and grafts
			
Instability	996.42	T84.02	Dislocation of internal joint prosthesis
		T84.020	Dislocation of internal right hip prosthesis
		T84.021	Dislocation of internal left hip prosthesis
		T84.028	Dislocation of other internal joint prosthesis
		T84.029	Dislocation of unspecified internal joint prosthesis
		M24.3	Pathological dislocation of joint, not elsewhere classified
		M24.35	Pathological dislocation of hip, not elsewhere classified
		M24.4	Recurrent dislocation of joint
		M24.45	Recurrent dislocation, hip
		S73.0	Subluxation and dislocation of hip
		S73.00	Unspecified subluxation and dislocation of hip
		S73.001	Unspecified subluxation of right hip
		S73.002	Unspecified subluxation of left hip
		S73.003	Unspecified subluxation of unspecified hip
		S73.004	Unspecified dislocation of right hip
		S73.005	Unspecified dislocation of left hip
		S73.006	Unspecified dislocation of unspecified hip
		S73.01	Posterior subluxation and dislocation of hip
		S73.011	Posterior subluxation of right hip
		S73.012	Posterior subluxation of left hip
		S73.013	Posterior subluxation of unspecified hip
		S73.014	Posterior dislocation of right hip
		S73.015	Posterior dislocation of left hip
		S73.016	Posterior dislocation of unspecified hip
		S73.02	Obturator subluxation and dislocation of hip
		S73.021	Obturator subluxation of right hip
		S73.022	Obturator subluxation of left hip
		S73.023	Obturator subluxation of unspecified hip
		S73.024	Obturator dislocation of right hip
		S73.025	Obturator dislocation of left hip
		S73.026	Obturator dislocation of unspecified hip
		S73.03	Other anterior subluxation and dislocation of hip
		S73.031	Other anterior subluxation of right hip
		S73.032	Other anterior subluxation of left hip
		S73.033	Other anterior subluxation of unspecified hip
		S73.034	Other anterior dislocation of right hip
		S73.035	Other anterior dislocation of left hip
		S73.036	Other anterior dislocation of unspecified hip
		S73.04	Central subluxation and dislocation of hip
		S73.041	Central subluxation of right hip
		S73.042	Central subluxation of left hip
		S73.043	Central subluxation of unspecified hip
		S73.044	Central dislocation of right hip
		S73.045	Central dislocation of left hip
		S73.046	Central dislocation of unspecified hip
			
Breakage	996.43	T84.01	Broken internal joint prosthesis
		T84.010	Broken internal right hip prosthesis
		T84.011	Broken internal left hip prosthesis
		T84.018	Broken internal joint prosthesis, other site
		T84.019	Broken internal joint prosthesis, unspecified site
			
Periprosthetic fracture	996.44	M97	Periprosthetic fracture around internal prosthetic joint
		M97.0	Periprosthetic fracture around internal prosthetic hip joint
		M97.01	Periprosthetic fracture around internal prosthetic right hip joint
		M97.02	Periprosthetic fracture around internal prosthetic left hip joint
		M97.8	Periprosthetic fracture around other internal prosthetic joint
		M97.9	Periprosthetic fracture around unspecified internal prosthetic joint
			
Aseptic loosening	996.41	T84.03	Mechanical loosening of internal prosthetic joint
		T84.030	Mechanical loosening of internal right hip prosthetic joint
		T84.031	Mechanical loosening of internal left hip prosthetic joint
		T84.038	Mechanical loosening of other internal prosthetic joint
		T84.039	Mechanical loosening of unspecified internal prosthetic joint
			
	996.45	T84.05	Periprosthetic osteolysis of internal prosthetic joint
		T84.050	Periprosthetic osteolysis of internal prosthetic right hip joint
		T84.051	Periprosthetic osteolysis of internal prosthetic left hip joint
		T84.058	Periprosthetic osteolysis of other internal prosthetic joint
		T84.059	Periprosthetic osteolysis of unspecified internal prosthetic joint
		M89.5	Osteolysis
		M89.55	Osteolysis, thigh
			
	996.46	T84.06	Wear of articular bearing surface of internal prosthetic joint
		T84.060	Wear of articular bearing surface of internal prosthetic right hip joint
		T84.061	Wear of articular bearing surface of internal prosthetic left hip joint
		T84.068	Wear of articular bearing surface of other internal prosthetic joint
		T84.069	Wear of articular bearing surface of unspecified internal prosthetic joint
			
Other	996.47	T84.09	Other mechanical complication of internal joint prosthesis
		T84.090	Other mechanical complication of internal right hip prosthesis
		T84.091	Other mechanical complication of internal left hip prosthesis
		T84.098	Other mechanical complication of other internal joint prosthesis
		T84.099	Other mechanical complication of unspecified internal joint prosthesis
	996.49	T84.4	Mechanical complication of other internal orthopedic devices, implants, and grafts
		T84.41	Breakdown (mechanical) of other internal orthopedic devices, implants and grafts
		T84.418	Breakdown (mechanical) of other internal orthopedic devices, implants and grafts
		T84.42	Displacement of other internal orthopedic devices, implants and grafts
		T84.428	Displacement of other internal orthopedic devices, implants and grafts
		T84.49	Other mechanical complication of other internal orthopedic devices, implants and grafts
		T84.498	Other mechanical complication of other internal orthopedic devices, implants and grafts
		T84.3	Mechanical complication of other bone devices, implants and grafts
		T84.31	Breakdown (mechanical) of other bone devices, implants and grafts
		T84.318	Breakdown (mechanical) of other bone devices, implants and grafts
		T84.32	Displacement of other bone devices, implants and grafts
		T84.328	Displacement of other bone devices, implants and grafts
		T84.39	Other mechanical complication of other bone devices, implants and grafts
		T84.398	Other mechanical complication of other bone devices, implants and grafts
			
	996.77	T84.8	Other specified complications of internal orthopedic prosthetic devices, implants and grafts
		T84.81	Embolism due to internal orthopedic prosthetic devices, implants and grafts
		T84.82	Fibrosis due to internal orthopedic prosthetic devices, implants and grafts
		T84.83	Hemorrhage due to internal orthopedic prosthetic devices, implants and grafts
		T84.84	Pain due to internal orthopedic prosthetic devices, implants and grafts
		T84.85	Stenosis due to internal orthopedic prosthetic devices, implants and grafts
		T84.86	Thrombosis due to internal orthopedic prosthetic devices, implants and grafts
		T84.89	Other specified complication of internal orthopedic prosthetic devices, implants and grafts
		T84.9	Unspecified complication of internal orthopedic prosthetic device, implant and graft

**Appendix Table 2:** ICD-9 and ICD-10 codes used for postoperative complications.

Category	ICD 9	ICD10
Acute Kidney Failure	584.5, 584.6, 584.7, 584.8, 584.9	N17.0, N17.1, N17.2, N17.8, N17.9
Cardiovascular	426, 426.6, 427.1, 427.31, 427.32, 427.5, 427.69, 427.89, 428.21, 428.31, 428.33, 428.9, 444.89, 785.51, 997.1	I442, I455, I472, I4891, I4892, I469, I493, I498, I5021, I5031, I5033, I509, I748, R570, I97710, I97790, I9788
CVA	997.02, 433, 434, 997.02, 430, 431, 432, 433, 434	I97.81, I97.82, I63, I97.81, I97.82, I60, I61, I62, I63
DVT	451.1, 451.2, 451.8, 451.9, 453.2, 453.4, 453.8, 453.6, 453.9, 451, 452, 453	I8010, I803, I809, I82220, I82409, J9589, I82.4, I82.81, I82.890, I82.90, I82.220, I80, I81, I82
General Infection	730.28, 730.98, 711.08, 41.3, 38.9, 41.6, 41.7, 785.52, 790.7, 995.91, 995.92, 998.51, 998.59, 999.31, 3849	M4620, M4630, M0008, M0018, M0028, M0088, B961, A41.9, B96.4, B96.5, R65.21, R78.81, A41.9, R65.20, K68.11, T80.219A, A41.59
MI	410, 410.01, 410.02, 410.1, 410.1, 410.11, 410.12, 410.2, 410.21, 410.22, 410.3, 410.31, 410.32, 410.4, 410.41, 410.42, 410.5, 410.52, 410.6, 410.61, 410.62, 410.7, 410.71, 410.72, 410.8, 410.82, 410.9, 410.91, 410.92, 410.81, 410	I2109, I2109, I2109, I2109, I2109, I2109, I2109, I2119, I2119, I2119, I2111, I2111, I2111, I2119, I2119, I2119, I2129, I2129, I2129, I2129, I2129, I214, I214, I214, I2129, I2129, I213, I213, I213, I2101, I2101, I2102, I2121, I21, I22
PE	415.0, 415.11, 415.13, 415.19, 415.1	I26.02, I26.09, I26.90, I26.92, I26.93, I26.94, I26.99, T84.81, I26
PNA	480, 480.1, 480.2, 480.8, 480.3, 480.9, 481, 482.2, 482, 482.1, 482.4, 482.41, 482.42, 482.49, 482.32, 482.39, 482.82, 482.83, 483, 482.89, 482.9, 483.1, 483.8, 485, 486, 507, 997.31, 997.32, 997.39	J12.0, J12.1, J12.2, J12.3, J12.81, J12.89, J12.9, J13, J14, J15.0, J15.1, J15.20, J15.211, J15.212, J15.29, J15.3, J15.4, J15.5, J15.6, J15.7, J15.8, J15.9, J16.0, J16.8, J18.0, J18.1, J18.8, J18.9, J69.0, J95.851
Pulmonary	511.9, 512.1, 518, 518.4, 518.5, 518.81, 997.3	J918, J95811, J9811, J9819, J810, J9600, J9620
Transfusion (PCS)	99.00, 99.01, 99.02, 99.03, 99.04, 99.05, 99.06, 99.07, 99.08, 99.09	30233N1, 30243N1, 30233H1, 30243H1, 30233P1, 30243P1, 30233N0, 30243N0, 30233H0, 30243H0, 30233P0, 30243P0, 3023, 3024, 3028
Wound	998.3, 998.31, 998.32, 998.33, 998.83, 998.11, 998.12, 998.13, 998.1, 998.3	T81.30, T81.31, T81.32, T81.33, M96.810, M96.811, M96.830, M96.831, M96.84, M96.842, M96.843, T81.3

**Appendix Table 3:** Propensity score matched analysis of index hospitalization complications for non-PJI patients.

	SOT patients	Matched controls	*P*-Value
Any Complication	42.0%	48.3%	.007
Transfusion	28.3%	36.4%	<.001
Cardiovascular	7.4%	9.1%	.172
MI	0.6%	0.6%	.999
CVA	0.3%	0.7%	.270
DVT	1.6%	1.4%	.723
PE	0.3%	0.3%	.852
Pneumonia	2.2%	2.3%	.942
Renal	10.8%	9.2%	.244
Wound	2.8%	3.1%	.662
Pulmonary	3.1%	5.1%	.041
Mortality	0.3%	0.2%	.833
LOS	5.0	6.0	<.001

**Appendix Table 4:** Propensity score matched analysis of index hospitalization complications for PJI patients.

	SOT patients	Matched controls	*P*-Value
Any Complication	79.7%	76.0%	.345
Transfusion	37.8%	45.0%	.148
Cardiovascular	14.7%	14.7%	.999
MI	0.7%	1.9%	.356
CVA	0.7%	1.9%	.356
DVT	5.6%	4.4%	.573
PE	0.7%	2.3%	.252
Pneumonia	5.6%	7.2%	.509
Renal	29.4%	23.8%	.180
Wound	9.1%	9.6%	.870
Pulmonary	8.4%	11.4%	.320
Mortality	2.1%	3.3%	.486
LOS	10.0	14.2	.010

## Supplementary data

Conflict of Interest Statement for All Authors
